# Encapsulation of Fullerenes: A Versatile Approach for the Confinement and Release of Materials Within Open-Ended Multiwalled Carbon Nanotubes

**DOI:** 10.3389/fbioe.2021.644793

**Published:** 2021-03-10

**Authors:** Stefania Sandoval, Gerard Tobias

**Affiliations:** Institut de Ciència de Materials de Barcelona (ICMAB-CSIC), Campus UAB, Barcelona, Spain

**Keywords:** carbon nanotubes, fullerenes, filling, corking, release

## Abstract

We have employed fullerenes as versatile agents to “cork” the open tips of multiwalled carbon nanotubes (MWCNTs), and as promoting species for the release of the inorganic material filled within the nanotubes’ cavities. High Z element compounds, namely, PbI_2_, ZnI_2_, and CeI_3_, were chosen to easily determine the presence of the filler inside the hosting nanotubes by transmission electron microscopy (TEM). Fullerenes can isolate inorganic nanostructures confined within the hollow cavities of MWCNTs, which allows the removal of the external material remnant after the filling. Otherwise, taking advantage of the affinity of fullerenes with selected solvents, we have confirmed the ability of the C_60_ molecules to promote the displacement of the inorganic guest from the host. We propose two different strategies to trigger the release, employing vapor and liquid phase treatments. The first protocol involves annealing filled MWCNTs in presence of fullerenes (to obtain C_60_PbI_2_@MWCNTs) and the subsequent washing of the sample in ethanol under mild conditions. On the other hand, the simultaneous introduction of the C_60_ molecules and the liberation of the guest are produced by a single step wet procedure; the latter being potentially useful when materials that are not stable at high temperatures are employed for filling.

## Introduction

The wide range of diameters of both single walled (SWCNTs) and multiwalled carbon nanotubes (MWCNTs) make their cavities susceptible of filling with diverse foreign species. The presence and nature of the guest material into the hollow cavities can alter the properties of the hosting template, improving its optical and electrical behavior (del Carmen Giménez-López et al., 2011). Moreover, the confinement into a small area might notably alter the morphology, chemical and structural characteristics of the guest, leading to the formation of new crystalline structures (Marega and Bonifazi, 2014; Sandoval et al., 2019). The hollow cavities of CNTs are useful not only as nanoscale templates for the synthesis of nanocomposites or nanostructures, but also provide an alternative toward isolating functional molecules from external environments, preventing any undesirable interaction with outer species that can modify the properties of the inner material or even produce the degradation of its structure.

Different approaches are employed to fill materials within CNTs, the strategy of synthesis depending on the physicochemical properties and stability of the filler (Ajayan and lijima, 1993; Ujjal et al., 2010; Kharlamova et al., 2012; Sauer et al., 2012). One dimensional nanowires of a wide number of inorganic compounds (Meyer et al., 2000; Sloan et al., 2002; Philp et al., 2003; Kitaura et al., 2009), metallic nanoparticles (Zhang et al., 2013) and organic species, such as β-carotene (Yanagi et al., 2006), small proteins and biomolecules (Guo et al., 1998), as well as graphene derivatives (Chuvilin et al., 2011) and fullerenes (Yudasaka et al., 2003) can be confined within the inner surface of CNTs. In this way, hybrid materials with diverse characteristics are obtained. These can be used in a myriad of applications, namely, molecular magnets (del Carmen Giménez-López et al., 2011), optoelectronics and photovoltaics (Zhou et al., 2015), battery electrodes (Prem Kumar et al., 2004), catalysis (Pan and Bao, 2008), or biomedicine (Klingeler et al., 2008; Martincic and Tobias, 2015).

However, a key factor for achieving a high filling yield is having CNTs with opened ends (Babaa et al., 2003). Regardless of the method used for the encapsulation of materials inside CNTs, an excess of the filling agent is typically employed (Dujardin et al., 1998; Sloan et al., 2002). Thus, an important amount of material remains outside the nanotubes after the filling step. The filling process is usually reproducible; nevertheless, the presence of external material hinders the quantification of the filling yield (Ballesteros et al., 2009). Furthermore, the removal of the non-encapsulated compounds is necessary to both allow a proper characterization of the sample and to determine how the inner material modifies the properties of the resulting nanocomposite. Otherwise, the properties of the sample cannot be exclusively attributed to the confined species but also to the presence of material external to the CNTs (Brown et al., 2003). An area in which filled carbon nanotubes have been extensively studied is in the biomedicine field (Martincic and Tobias, 2015). However, applications for *in vivo* imaging, drug delivery or tumor targeting require the absence of species remnant from the filling process, usually attached to the outer surface of the CNTs (Ge et al., 2017; Wang et al., 2020).

The easiest procedure to clean external material from the sample involves the use of solvents that, in general, are also capable to dissolve the filling agent. Therefore, unless the encapsulated material has a strong interaction with the CNTs, this approach not only removes the external compounds but also washes out the confined nanostructure, since the ends of the CNTs are opened (Shao et al., 2006). In the case of single walled carbon nanotubes (SWCNTs), it has been reported that the ends can be closed by high temperature treatments (ca. 900°C) (Geng et al., 2004); thus, allowing the removal of the external material whilst preserving the encapsulated compounds (Shao et al., 2006). In case of MWCNTs, closing their ends by high temperature annealing is much more difficult and requires the formation of C-C bonds generating high curvature strain (Mazzoni et al., 1999). Moreover, due to the presence of a larger cavity, a much higher energy and hence, much higher annealing temperatures than SWCNTs are necessary to induce the closing (Martincic et al., 2019). Despite the later protocol demonstrated to be highly efficient for the formation of hermetically closed nanocapsules, a wide range of substances can be decomposed at high temperature and an alternative strategy for sealing up the ends of the nanotubes is required. Capobianchi et al. proposed the impregnation of the open ended filled MWCNTs with a solvent unable to solubilize the filling agent when entering into the hollow cavity by capillarity. Afterward, a washing solvent could be added to the mixture without affecting the inner material (provided both liquids present a low miscibility) (Capobianchi et al., 2007). However, the resulting filling yield is low and the elimination of the protecting solvent could be problematic.

Fullerenes, also called buckyballs, are composed entirely of carbon arranged in hexagonal and pentagonal rings (resembling the classic soccer balls), forming of a hollow sphere (Nessim, 2010). Taking advantage of the strong affinity of fullerenes to enter into the inner cavities of SWCNTs, these molecules have also been employed as corking agents for the containment of materials previously confined within their cavities (Shao et al., 2008; Ren and Pastorin, 2008). Sloan et al. (2000) showed that the presence of fullerenes within the hollow cavity of SWCNTs prevents the introduction of other foreign materials and a pH triggered release of materials from SWCNTs has been achieved using functionalized fullerenes as corks (Luksirikul et al., 2010).

The preparation of C_60_@SWCNTs; usually called nanopeapods (NPPs) has been widely studied. The interaction mechanisms between the nanotubes and fullerenes involved in the filling process, as well as the behavior of the C_60_ upon encapsulation, have attracted much interest due to the particular structures that can be formed (Warner et al., 2008). Theoretical studies have shown that, under the appropriate energetic conditions, fullerenes could be initially adsorbed onto the external walls prior to encapsulation (Berber et al., 2002), and coalesce after confinement (Tang et al., 1999; Hernández et al., 2003). Besides, SWCNTs with the appropriate diameter are able to perfectly accommodate a single molecule of C_60_ within their two walls (Nikolaev et al., 1997). Thus, their proximity to the inner surface of the nanotubes allows a strong interaction between the buckyball and the nanotube. In case of fullerenes’ encapsulation into MWCNTs, both theoretical and experimental studies involve considerations that are more complex and have been barely described. The successful encapsulation and stability of the resulting NPPs might be strongly affected by the diameter of the host, being closely linked not only to the surface interaction of fullerenes and the inner walls of the nanotubes, but also to the configuration adopted by the particles inside the tubes and the mutual interaction between them. It has been reported that wider inner diameters allow the introduction of a larger amount of fullerenes, with accommodations within the MWCNTs cavities ranging from zig-zag chains to irregular arrangements (Fröhlich et al., 2004), tending to agglomerate and cluster (Maggini et al., 2014).

Another important issue usually considered for some of the potential applications of filled nanotubes is the controlled release of the encapsulated material. This process requires breaking energy barriers that can be present due to attractive interactions established between the inner structures and the hosting CNTs after filling (Gao et al., 2003). Releasing and transport mechanisms of liquid (Longhurst and Quirke, 2007) or gaseous substances (Wang, 2009), as well as the assisted removal of the filled materials have been explored (Král and Tománek, 1999; Insepov et al., 2006; Longhurst and Quirke, 2007; Panczyk et al., 2013). Considering the high affinity and strong intermolecular forces that can exist between fullerenes and CNTs, theoretical studies on the capability of fullerenes for displacing different species from the cavities of SWCNTs have been carried out (Xue et al., 2012; Saikia et al., 2013).

Previous studies have demonstrated the enormous potential of MWCNTs as isolating agents and carriers of materials with high interest in the field of biomedicine. Considering the limitations still present when preparing clean and hermetically closed MWCNTs-based nanocapsules, the aim of this study was to evaluate the capability of fullerenes to act not only as corking agents to preserve the integrity of the guest molecules confined within MWCNTs, but also their potential as release agents to promote the controlled liberation of the guest molecules. Two different approaches, involving annealing and wet treatments at room temperature, were tested in order to provide an alternative for obtaining nanocapsules. These approaches are compatible with materials unstable at high temperatures.

## Materials and Methods

### MWCNTs Purification

Chemical vapor deposition MWCNTs (Thomas Swan & Co., Ltd.) were steam treated during 5 h at 900°C, in order to remove amorphous carbon and graphitic nanoparticles and to open their ends (Cabana et al., 2015). Subsequently, the sample was treated with a 6 M HCl solution to remove the metal nanoparticles exposed after the annealing treatment (Ballesteros et al., 2008). The obtained dispersion was filtered, rinsed with distilled water until neutral pH and dried overnight at 60°C.

### Filling of MWCNTs by Molten Phase Procedure

PbI_2_@MWCNTs were prepared employing 6 mg of purified MWCNTs and 140 mg of PbI_2_ (Strem Chemicals Inc.). In an argon-filled glove box both, PbI_2_ and MWCNTs were ground together with an agate mortar and pestle until the mixture presented a uniform color. Afterward, the powder was transferred into a silica ampoule, evacuated and sealed under vacuum. The ampoule was placed into a furnace, where it dwelled at 500°C (temperature above the melting point of the salt) during 12 h. Finally, the sample was cooled at room temperature and then it was opened under inert atmosphere. ZnI_2_@MWCNTs and CeI_3_@MWCNTs were prepared following the same protocol; the temperature of treatment being selected taking into account the melting point of the selected materials. The ZnI_2_/MWCNTs mixture (300 mg/10 mg) was annealed at 475°C, while the CeI_3_@MWCNTs resulted from annealing 10 mg of MWCNTs in presence of 200 mg of CeI_3_ at 900°C. Both ZnI_2_ (99.99%) and CeI_3_ (99.99%) were purchased from Sigma-Aldrich.

### C_60_ Corking Into the Opened-Ended Metal Halide Filled MWCNTs (MX@MWCNTs)

C_60_ close-ended filled MWCNTs were prepared by grinding both, fullerenes (C_60_, 99.5%, SES Research) and MWCNTs previously filled with PbI_2_, ZnI_2_ or CeI_3_. Different ratios of filled CNTs and fullerenes were employed. The corking was carried out by annealing the mixture at 400°C, during 48 h, under vacuum (inside a silica ampoule) (Hirahara et al., 2000).

### Washing of the External Material

The material deposited on the external surface of the MWCNTs was removed by sonicating the samples (5 mg) in 30 mL of distilled water for 15 min and refluxing during 24 h at 100°C. Finally, the sample was recovered by filtration employing a 0.2 μm polycarbonate membrane, rinsed with distilled water and the procedure was repeated. The recovered powder was dried at 60°C overnight.

### PbI_2_ Release Assisted by Ethanol/C_60_

#### Protocol 1

Five-mg of the C_60_PbI_2_@MWCNTs sample were suspended in absolute ethanol (Panreac, max 0.02% water) and dispersed by sonication during 15 min. Afterward, the mixture was refluxed (80°C) during 16 h, cooled down and filtered using a 0.2 μm polycarbonate membrane. After drying overnight at 60°C, the material was characterized using TEM.

#### Protocol 2

Five-mg of fullerenes were suspended in pure ethanol and dispersed by sonication during 30 min. Afterward, 5 mg of the PbI_2_@MWCNTs were added to the dispersion and sonicated for 15 min. The mixture was refluxed (80°C) during 16 h, cooled down and filtered using a 0.2 μm polycarbonate membrane. After drying overnight at 60°C, the material was characterized using TEM.

### Characterization

The filling of the samples was evaluated by means of transmission electron microscopy (TEM) and scanning electron microscopy SEM, while their composition was determined by energy dispersive X-ray (EDX) analysis. TEM images were acquired using a JEOL Jem 1210 electron microscope operating at 120 kV and a FEI Tecnai G2 F20 microscope (High resolution microscope-HRTEM) operating at 200 kV. SEM images and EDX analyses were performed using a QUANTA FEI 200 FEG-ESEM microscope operating at 20.0 kV. Samples were prepared by sonication of a small amount of the powder in anhydrous hexane (95%, Sigma-Aldrich). Afterward, the solution was placed, dropwise, onto a lacey carbon support grid and let to dry. Diffraction patterns were obtained in a Siemens D5000 diffractometer (Kα Cu). 2θ values were acquired at 0.02° intervals between 5° and 60°. Raman spectra were recorded using a Horiba Jobin Yvon operating at 532 nm and using 50 × objective. Acquisition time was set to 10–30 s and laser power to 0.5 mW. Spectra were recorded in the 100–3,000 cm^–1^ range, from different spots of the powdered samples.

## Results

### Microscopic Analysis of PbI_2_ Filled MWCNTs and C_60_ Corked PbI_2_@MWCNTs

**FIGURE 1:**
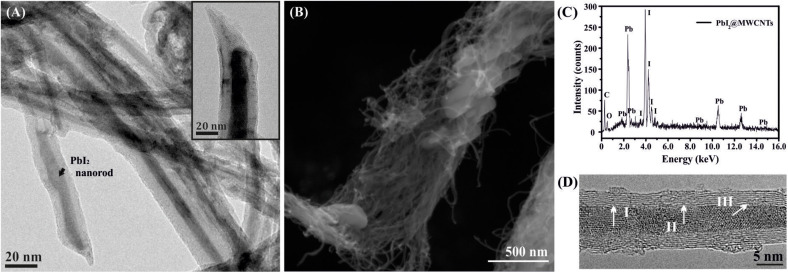
**(A)** TEM of PbI_2_ filled MWCNTs after molten phase capillary wetting synthesis; the inset shows a detail of an open ended PbI_2_@MWCNT. **(B)** SEM showing the morphology of the sample and **(C)** the corresponding EDX spectrum, confirming the presence of both Pb and I in the sample. **(D)** HRTEM of a MWCNT filled with polycrystalline PbI_2_; d-spacings of PbI_2_ determined *via* intensity profiles along the indicated white lines correspond to I = 3.5 Å, II = 3.3 Å, and III = 3.5 Å.

The inorganic material present inside the cavities of MWCNTs is easily distinguishable from CNTs when these are observed by TEM, which was employed to verify the encapsulation of PbI_2_ within the MWCNTs cavities. Figure 1A shows a low magnification image of PbI_2_@MWCNTs. A magnification of an open-ended PbI_2_@MWCNTs is observed in the inset. A SEM image of the PbI_2_@MWCNTs is included in Figure 1B, where the characteristic tubular morphology of the hosting nanostructure along with external PbI_2_ can be observed. The corresponding EDX spectrum is shown in Figure 1C. A *ca.* 3.5 Å lattice spacing was calculated from intensity profile analysis in selected regions of PbI_2_ filled MWCNTs [(Figure 1D), HRTEM micrographs].

**FIGURE 2:**
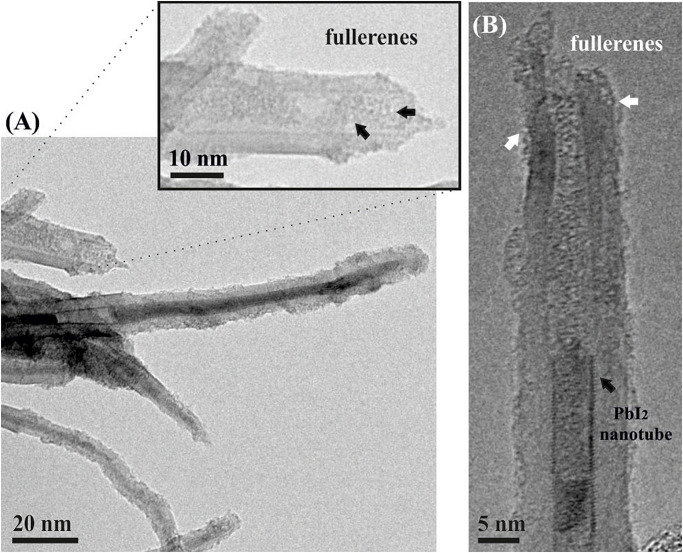
**(A)** C_60_PbI_2_@MWCNTs sample. A magnified area of an open ended CNT containing fullerenes is presented. **(B)** PbI_2_ filled nanotube with fullerenes corking and isolating the inorganic nanostructure; fullerenes can be also seen on the external walls of CNTs (pointed by white arrows).

400°C treatment of an open ended PbI_2_@MWCNTs/fullerenes mixture lead to the formation of C_60_PbI_2_@MWCNTs (Figures 2A,B). The presence of the inner nanorods within the hosting nanotubes has not been affected by the interaction of fullerenes with the sample, despite their affinity with the CNTs. TEM micrographs show that fullerenes are located in the inner surface of the nanotubes, blocking the open ends. Besides, a large number of C_60_ molecules are observed along the external walls of the hosts.

### Washing and Release Strategies

**FIGURE 3:**
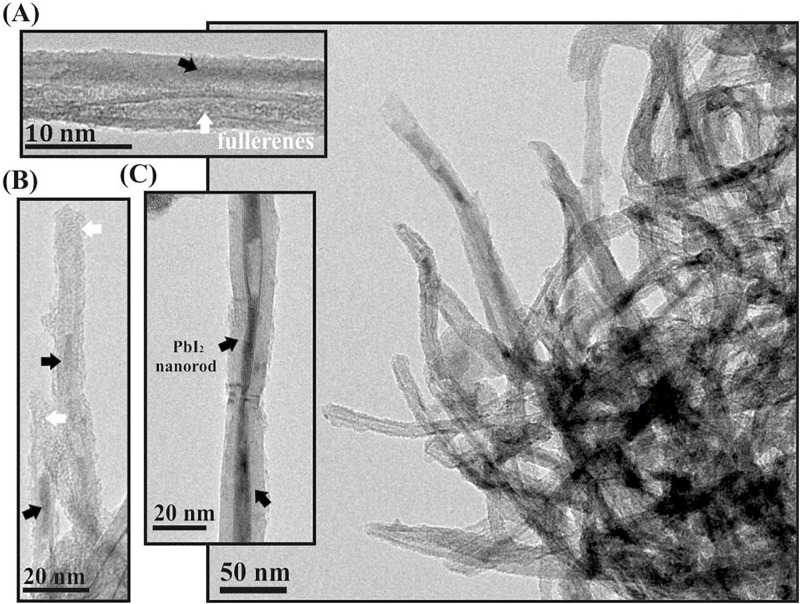
**(A–C)** show individual nanotubes containing both fullerenes (white arrows) and PbI_2_ nanostructures (black arrows) that have not been removed after washing. A low magnification image (right) shows that most of the corked nanotubes maintained their filling after washing.

After multiple washings of open-ended PbI_2_ filled MWCNTs with hot water, the inorganic salt was removed from the inner surface of the hosting nanotubes (Supplementary Figure 1). Otherwise, fullerenes present in the tips avoid releasing the PbI_2_ when C_60_PbI_2_@MWCNTs are subjected to the same protocol of washing (Figure 3). The proposed corking approach demonstrated to be versatile since allows the successful confinement of other inorganic salts, namely, ZnI_2_ and CeI_3_, after washing the external material from the outer surface of the nanotubes (Supplementary Figure 2).

**FIGURE 4:**
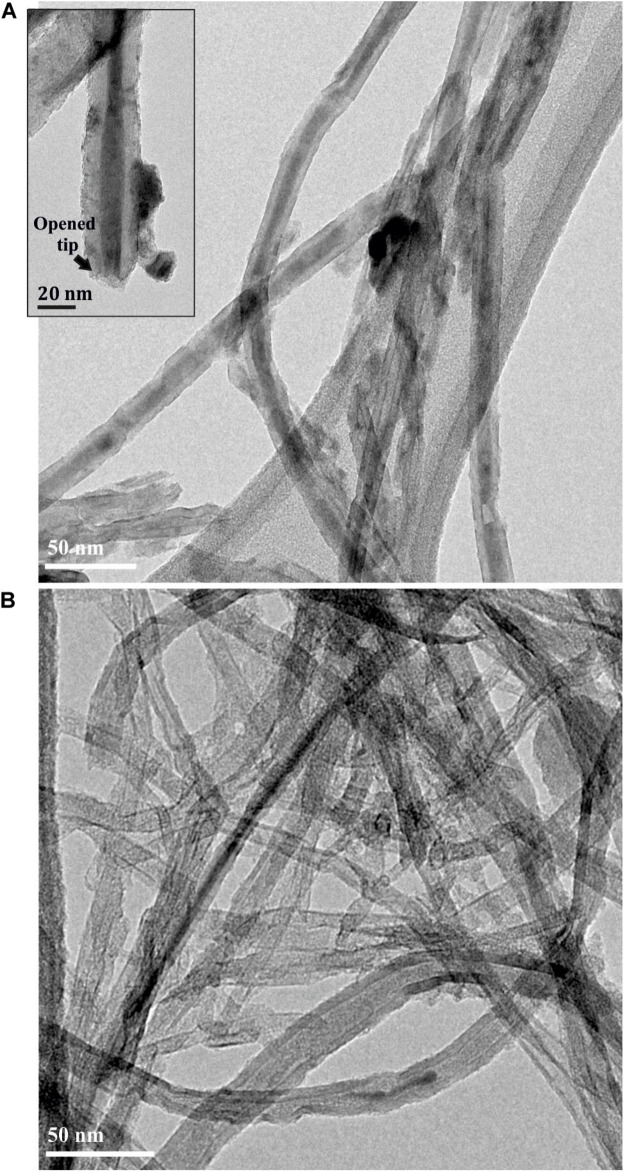
**(A)** PbI_2_@MWCNTs and **(B)** C_60_PbI_2_@MWCNTs after washing with anhydrous ethanol at 80°C during 16 h.

When ethanol, a solvent with affinity to the fullerenes was employed for washing (16 h under reflux), the C_60_ molecules located in the tips of the MWCNTs act as adjuvants of the displacement of the inorganic salt filled into the nanotubes (C_60_PbI_2_@MWCNTs sample). In absence of fullerenes (PbI_2_@MWCNTs) the release of PbI_2_ from the hosting nanotubes in presence of ethanol was not observed (Figures 4A,B). Removal of PbI_2_ from PbI_2_@MWCNTs was also triggered using a liquid phase approach under mild conditions of reaction. By suspending the sample in a C_60_ ethanol dispersion and refluxing, the inorganic salt was removed from the inner surface of the nanotubes and replaced by the fullerenes present in the medium (Figures 6A,B).

### XRD and Raman Spectroscopy of Filled Samples

XRD analysis of C_60_ZnI_2_@MWCNTs (Supplementary Figure 3) shows the characteristic diffraction pattern of C_60_, which signal induces the attenuation of the broad diffraction peak of MWCNTs (002, 2θ∼26°). Raman spectra (Supplementary Figure 4) of samples resulting of wet treatment using EtOH/C_60_ mixtures of both empty and PbI_2_ filled MWCNT confirm the presence of fullerenes in the material.

## Discussion

### End Corking of MWCNTs With Fullerenes

PbI_2_ is an interesting compound because is a large bandgap 2D layered material that has potential for semiconductor applications (Sinha et al., 2020) and, bearing heavy elements, could also be employed as contrast agent (Hernández-Rivera et al., 2017). PbI_2_ along with the other metal halides (ZnI_2_, CeI_3_) employed in this study are layered structures, which can lead to the formation of tubular van der Waals heterostructures when confined within the cavities of MWCNTs (Cabana et al., 2014; Sandoval et al., 2017; Sandoval et al., 2018). Besides, since the contrast in TEM imaging is highly dependent on the atomic/molecular weight of the material, PbI_2_ was chosen as a model compound (both elements, Pb and I, present a high atomic number) for filling MWCNTs. Thus, the inner grown nanorods composed by the relatively heavy atoms are easily distinguished from the CNTs when characterized by TEM (Figure 1A). SEM confirms that the morphology of MWCNTs remains unaltered after the filling experiment and also reveals the presence of PbI_2_ crystals external to the CNTs (Figure 1B). As expected, EDX analysis (Figure 1C) indicates the presence of Pb and I in the sample. Different d-spacings of the PbI_2_ crystallites present inside the MWCNTs cavities have been measured from the HRTEM images (Figure 1D). A spacing of *ca.* 3.5 Å is observed, in agreement with crystallographic data of bulk PbI_2_ [(002) reflection plane].

PbI_2_ filled MWCNTs were employed to evaluate the capability of fullerenes of blocking the open ends of CNTs. For this purpose, C_60_PbI_2_@MWCNTs were prepared from PbI_2_@MWCNTs by a vapor-phase method at 400°C, during 48 h (Tang et al., 1999). Temperatures between 300 and 450°C are considered to provide the energy necessary for the formation of NPPs (Ajayan and lijima, 1993) and *in situ* studies have detected mobility of fullerenes along the CNTs walls at ca. 325°C, followed by the entrance of fullerenes within the SWCNTs at 350°C (Hernández et al., 2003). Furthermore, the sublimation of C_60_ molecules, which is necessary for the vapor-phase encapsulation (Nikolaev et al., 1997), has been reported to start at relatively low temperatures (ca. 375°C), being favored under low pressure conditions (Sloan et al., 2000). Taking into account that the probability of C_60_ entering into the nanotubes decreases with the temperature, higher temperatures of treatment were not considered for this study. Figure 2A shows a low magnification TEM image of a C_60_PbI_2_@MWCNTs sample. As shown, the employed protocol allows the introduction of C_60_ molecules in the cavities of the MWCNTs that remained empty after the filling of the tubes with PbI_2_. The inset shows a magnification where the presence of fullerenes contained in an open-ended MWCNT is appreciated. Additionally, a HRTEM image of a MWCNT containing both, a PbI_2_ nanotube and fullerenes blocking the opened tips is presented in Figure 2B. The presence of fullerenes on the external walls of the nanotubes (pointed by white arrow) is in agreement with theoretical calculations that suggest an “optimum” trajectory of the C_60_ molecules when approaching to the CNTs. According to the studies of Berber et al. (2002), fullerenes may be initially physisorbed on the outer wall of the nanotube and subsequently diffuse along the CNT surface. In this way, the corking process involves an initial non-covalent functionalization of the CNTs walls with the C_60_ molecules before the filling. Afterward, fullerenes can displace either through the defects of the CNTs walls or *via* the opened tips, establishing strong electrostatic interactions with the inner surface of the nanotubes.

The most common methodology for the elimination of the excess of material present on the external surface of the hosting nanotubes consists in carrying out consecutive washings with a specific solvent, capable of solubilizing the guest specimens (Kierkowicz et al., 2017). PbI_2_ is relatively soluble in hot water, which can be employed to remove the external material present after the synthesis of PbI_2_@MWCNTs. Nevertheless, since the ends of the CNTs are opened, the process also washes out the material contained inside the nanotubes, and only empty MWCNTs can be seen in the sample (Supplementary Figure 1). The strong electrostatic interactions existing between the CNTs walls and the C_60_ molecules, combined with the poor solubility of fullerenes in water (Heymann, 1996), produce the corking of the opened tips of the hosting carbon nanotubes. Thus, C_60_ molecules avoid the removal of the inner nanostructures, while the external material is eliminated. For this reason, when the same protocol of washing (using hot water) is employed for the C_60_PbI_2_@MWCNTs, an important amount of PbI_2_ remained in the hosting cavity of the nanotubes (Figure 3). Images of individual CNTs containing PbI_2_ nanorods (pointed by black arrows) as well as fullerenes (white arrows) are also included (Figures 3A–C). The presence of PbI_2_ inside the MWCNTs confirms that fullerenes not only are useful for the isolation of inorganic material inside SWCNTs (Tobias et al., 2010), but also can be employed for the confinement of compounds within tubular carbon nanostructures with larger diameters (MWCNTs).

The optimum amount of fullerenes necessary to cork the CNTs was explored. Since an important amount of filling agent remains outside the nanotubes, the quantification of the CNTs/C_60_ ratio is not possible. However, the amount of fullerenes was selected in function of the mass of CNTs that was initially mixed with the inorganic salt. Thus, the totality of the sample after the endohedral functionalization with PbI_2_ (PbI_2_@MWCNTs) was mixed in 1:1, 1:2, 1:3, and 1:10 CNTs/C_60_ ratios. After treatment with the lowest amount of fullerenes (1:1 ratio), an important fraction of C_60_ was observed along the CNTs walls and tips. However, the amount of filled nanotubes decreased considerably after washing with water. Meanwhile, when the samples were treated with two and three parts of fullerenes, and subsequently washed, a higher frequency of filled nanotubes was observed. Finally, the treatment with the highest amount of fullerenes (1:10 CNTs/C_60_) did not show a significant variation in the frequency of PbI_2_@MWCNTs, in comparison with the sample annealed in presence of three parts of fullerenes. Since the use of the highest amount of C_60_ did not result in an increase of PbI_2_ filled nanotubes after washing, a 1:3 ratio (filled sample: fullerenes) appears to be the highest suitable ratio to be employed for corking.

In order to confirm that the introduction of C_60_ molecules within the filled CNTs is independent on the filling agent, MWCNTs filled with ZnI_2_ and CeI_3_ were also treated with fullerenes. The solubility of ZnI_2_ and CeI_3_ in water in normal conditions is considerably high (Lyde et al., 2003-2004), and the external material is thus easily removable by simple washings with aqueous solutions. After washing, the presence of both ZnI_2_ and CeI_3_ nanorods encapsulated within the MWCNTs is observed (Supplementary Figure 2). After XRD analysis of the washed C_60_ZnI_2_@MWCNTs (Supplementary Figure 3, continuous red line) the 002 diffraction peak (d-space 3.4 Å at ca. 26°), characteristic from MWCNTs is strongly attenuated by the presence of C_60_, which diffraction pattern can be attributed to the Fm3m fcc lattice (Zhou et al., 2004; Ginzburg et al., 2005). In this way, the use of fullerenes is presented as a useful protocol to isolate inorganic halides grown within MWCNTs, which allows the removal of impurities remnant after the filling process.

### Release of Crystalline Structures From MWCNTs Assisted by Fullerenes

The use of fullerenes to assist on the displacement of inorganic encapsulated nanostructures, employing ethanol as promoting solvent, was additionally explored. If the physicochemical properties of fullerenes are considered, employing a solvent with certain affinity with the C_60_ molecules may facilitate their mobility inside the CNTs. These species could act as appropriate replacing agents favoring the displacement of the inorganic nanomaterial and triggering its release from the host. For this purpose, the C_60_PbI_2_@MWCNTs were dispersed and refluxed in ethanol during 16 h. PbI_2_ is not soluble in ethanol (Lyde et al., 2003-2004) and under normal conditions (in absence of fullerenes), the inorganic PbI_2_ confined within the hosting cavity should be retained in the MWCNTs. Thus, PbI_2_@MWCNTs was additionally washed with ethanol as control. The obtained samples were characterized employing TEM imaging. Figure 4A shows a low magnification TEM image of the PbI_2_@MWCNTs after washing with ethanol. As expected, the solvent was unable to remove PbI_2_ from the interior of the MWCNTs despite being exposed to the solvent through the opened tips (inset), due to the low solubility of the PbI_2_ in ethanol. In contrast, when the sample was previously annealed in presence of fullerenes and subsequently washed using the same conditions, the majority of the inorganic guests were washed out from the MWCNTs (Figure 4B), indicating that in this case fullerenes foster the release of the encapsulated compounds.

The entrance of the C_60_ molecules into the nanotubes requires specific energies (Berber et al., 2002), which can be provided either by high temperature treatments (Smith et al., 1998), or by the assistance of a solvent with certain characteristics (Yudasaka et al., 2003). Yudasaka et al. (2003) proposed a successful methodology, which they called “nano-extraction,” consisting in the incorporation of fullerenes within SWCNTs, promoted by the suspension of a mixture of both materials in ethanol. This liquid phase technique, leads to the formation of NPPs and takes advantage of the solubility of the C_60_ molecules in the solvent, which although poor, is strong enough to promote the interaction between the fullerenes and the CNTs, provoking the incorporation of the C_60_ molecules into the nanotubes.

One could think that the strong non-covalent interactions (Okada et al., 2001) formed between the C_60_ molecules and the CNTs walls should constitute a highly energetic barrier to be overcome in order to produce the mobility of the fullerenes along the inner cavity of the nanotube. Additionally, electrostatic interactions, such as van der Waal forces, existing between the inorganic nanostructure and the CNTs may be an additional difficulty toward the displacement of the guests outside the nanotubes. Theoretical studies have described the energetic stability of C_60_ encapsulated within a SWCNT (nanopeapod, NPP), including the high binding energies present between the fullerenes and the nanotube (Dubay and Kresse, 2004). The strength and stability of these interactions depends on the symmetry and dimensions of the nanotube. Meanwhile, the release process requires the use of a solvent in which fullerenes are highly soluble (Simon et al., 2007). Fan et al. (2007) studied the effect of the diameter in both the incorporation of fullerenes into SWCNTs and their release assisted by toluene, finding that small diameters of the host favor the encapsulation, while the removal of the encapsulated C_60_ molecules presents the opposite trend. For a system formed by MWCNTs and fullerenes, a theoretical approach would be more complex. In fact, the incorporation of fullerenes within MWCNTs has been barely reported (Kondo et al., 2003).

Considering that the diameter and the contact surface between the nanotubes and the C_60_ molecules may play a role in the release of the inorganic material, fullerenes incorporated within the cavities of MWCNTs should possess weaker affinity with the inner walls if compares to SWCNTs. Nevertheless, the established interactions appear to still be strong and the trafficking of the fullerenes along the cavities of the nanotubes may also require a driving force. According to our observations, the attraction forces between the C_60_ molecules and the inner walls are significantly altered when fullerenes are confined within MWCNTs. This is in agreement with theoretical studies on SWCNTs of different dimensions filled with drug molecules, where an increase in the release of the inner material is observed for nanotubes with higher diameters (Saikia et al., 2013).

**FIGURE 5:**
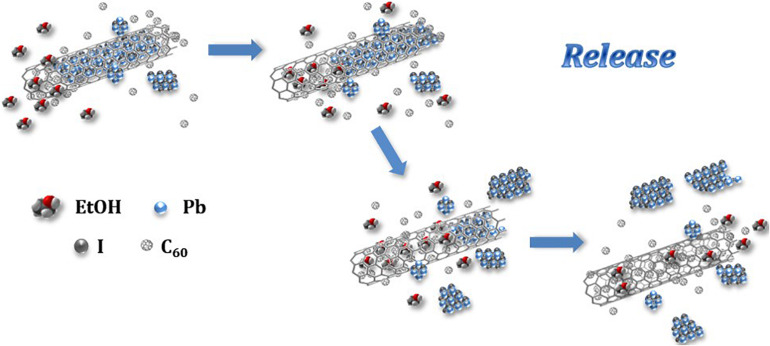
Schematic representation of the fullerene assisted release of PbI_2_ from MWCNTs employing ethanol as promoting solvent.

A graphical representation of the process of release of the inorganic salt from the MWCNTs cavities is presented in Figure 5. A competitive replacement of the inorganic salt fraction (bluish crystals) contained within the tube is produced. The high diffusivity of the liquid solvent (EtOH) contributes toward the mobility of the fullerenes within the MWCNT, expelling out the inorganic salt. Additionally, the increase in the contact area between the fullerenes and the CNT wall might enhance the stability of the system by the creation of van der Waal forces between the hosting nanotubes and the C_60_ molecules (Xue et al., 2012).

**FIGURE 6:**
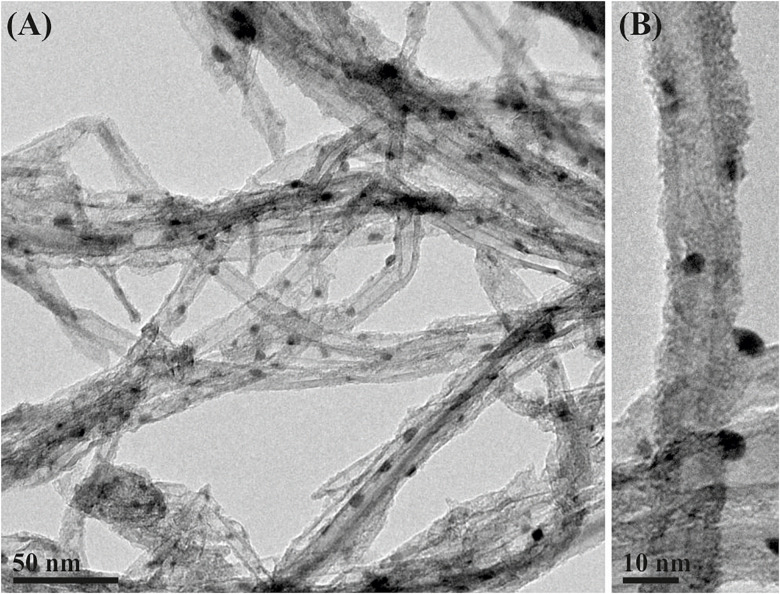
**(A)** PbI_2_@MWCNTs after washing at 80°C during 16 h with a dispersion of fullerenes in absolute ethanol. **(B)** Detail of a PbI_2_@MWCNT after washing with an EtOH/C_60_ mixture.

Considering that many materials susceptible of being filled within the cavities of CNTs are not stable at high temperatures, and those other approaches, different from the molten phase capillary wetting or vapor-phase reactions are necessary for their incorporation inside the nanotubes and the subsequent C_60_ corking; we applied an alternative strategy for the release process. Employing the nano-extraction technique (Yudasaka et al., 2003) to the removal of the inner structure assisted by fullerenes, the process not only was reduced to a single step, but also allowed the insertion of fullerenes at mild conditions, having potential applications to the release of non-thermally stable guest nanostructures. By suspending PbI_2_@MWCNTs in a dispersion of fullerenes in pure ethanol and subsequently refluxing the mixture, we have been able to remove the inorganic halide from the inner cavities of the MWCNTs. A low magnification image of the PbI_2_ filled MWCNTs after washing with the ethanolic dispersion of fullerenes is presented in Figure 6A. Microscopy analyses confirm the replacement of the encapsulated PbI_2_ by the C_60_ molecules. The small nanoparticles observed on the outer surface of the nanotubes correspond to the PbI_2_ displaced from the cavities of CNTs. The latest protocol results in a higher amount of fullerenes present along the nanotubes. C_60_ molecules were homogeneously distributed through both the external walls of the nanotubes and inside their cavities (Figure 6B). Raman spectra of both PbI_2_@MWCNTs before and after washing with ethanol are presented in Supplementary Figure 4. C_60_ alone and MWCNTs before and after introducing fullerenes within their cavities (by dispersing them in a C_60_/ethanol mixture) are included for comparison. In all cases (except for C_60_) the characteristic D (1,341 cm^–1^, out of plane vibration) and G (1,585 cm^–1^, stretching of sp^2^ bonds from the graphitic structure) bands of graphitic-based materials are observed. After treatment of both empty and PbI_2_ filled MWCNTs in presence of C_60_, the signal arising from MWCNTs is attenuated and the most prominent peak, corresponding to the pentagonal pinch mode Ag(2) of fullerenes (1,461.7 cm^–1^) appears, confirming their presence in the analyzed powder.

**FIGURE 7:**
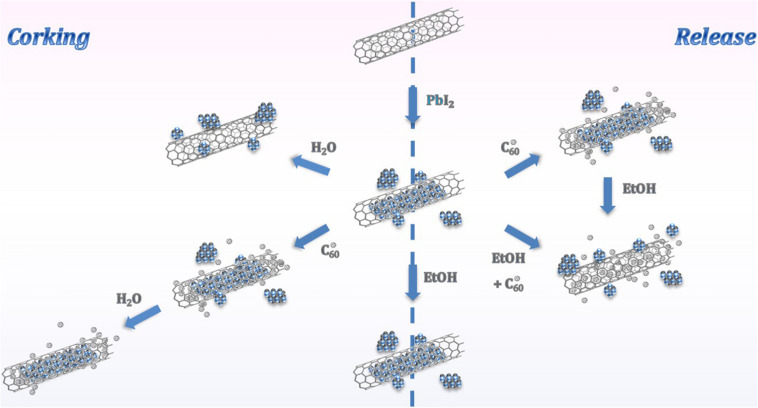
Schematic representation of the mechanisms of corking of MWCNTs and release of the guest material from the hosting nanotubes promoted by fullerenes.

Figure 7 summarizes the proposed complementary approaches: corking of MWCNTs and release of inner material confined within their cavities. On the one hand, fullerenes can be employed as efficient corking agents of open-ended MWCNTs, which facilitates the removal of external material resulting from the filling process (left). This protocol is useful as long as the cleaning of the filled nanotubes is carried out employing solvents which affinity with the C_60_ molecules is extremely low. On the other hand a versatile protocol for the removal of inorganic material from the inner cavities of MWCNTs is proposed (right). The encapsulation of fullerenes, followed by the ethanol assisted migration of the C_60_ molecules, arises as a potential strategy to trigger the liberation of materials. Otherwise, a liquid phase methodology, involving a single step procedure and mild conditions is suggested for the simultaneous incorporation of fullerenes and release of compounds from the hosting CNTs.

## Conclusion

We have studied the use of fullerenes, either to isolate inorganic materials present in the cavities of MWCNTs, or to promote the release of the guest structures. Fullerenes act as corking agents, as long as the cleaning of the filled nanotubes is carried out with solvents which affinity with the C_60_ molecules is very low. The presence of fullerenes avoids the release of the encapsulated payloads during the removal of the external non-filled inorganic material remnant after the filling procedure. Otherwise, fullerenes are useful to trigger the liberation of guest structures from the MWCNTs cavities when solvents with considerable affinity to the C_60_ molecules are employed to promote the release. In the present study, ethanol was employed as promoting solvent to favor the mobility of the C_60_ molecules within MWCNTs, assisting the removal of inorganic nanostructures previously grown within the hosting nanotubes. A versatile protocol, involving high temperature treatments and liquid phase techniques has been proposed to induce the liberation of guest structures from the cavities of MWCNTs. We believe that this approach can be employed for a large variety of both organic and inorganic compounds, opening up new possibilities for their containment and controlled release from carbon nanotubes.

## Data Availability Statement

The raw data supporting the conclusions of this article will be made available by the authors, without undue reservation.

## Author Contributions

SS and GT designed the experiments, analyzed the data, and wrote the manuscript. SS performed the experiments. Both authors have given approval to the final version of the manuscript.

## Conflict of Interest

The authors declare that the research was conducted in the absence of any commercial or financial relationships that could be construed as a potential conflict of interest.
