# Supplementary material for: Encapsulation of Fullerenes: A Versatile Approach for the Confinement and Release of Materials Within Open-Ended Multiwalled Carbon Nanotubes
Source: Front Bioeng Biotechnol. 2021 Mar 10;9:644793. doi: 10.3389/fbioe.2021.644793 (PMC7987908; doi:10.3389/fbioe.2021.644793)
Supplement: Supplementary file 1 [file Data_Sheet_1.pdf]

Supplementary Material

Supplementary Figures

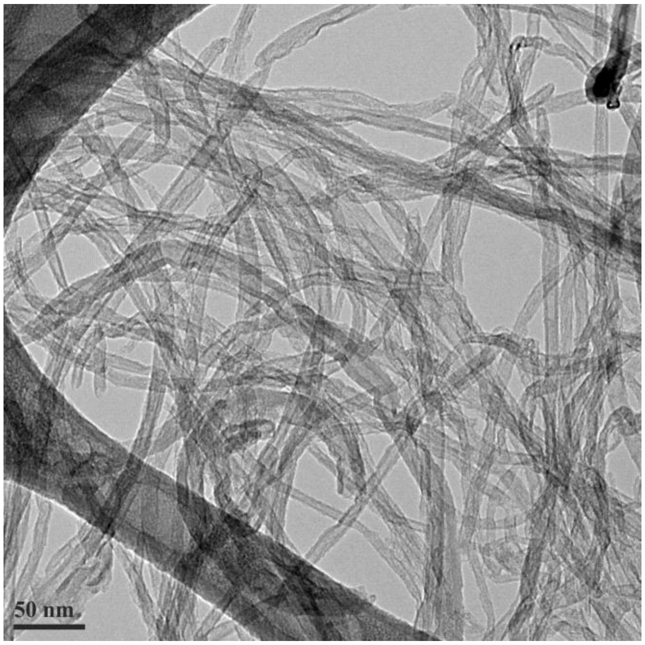

Supplementary Figure 1. Low magnification image of the PbI_2@MWCNTs after washing with water.

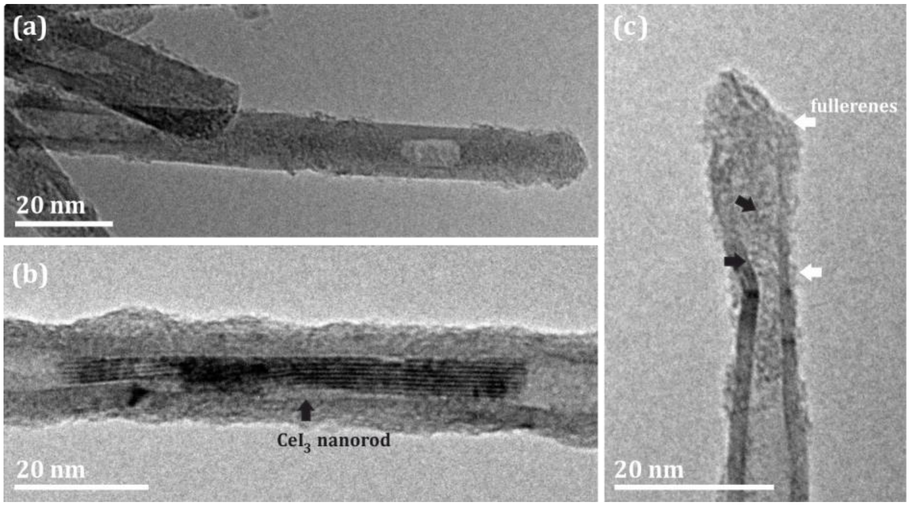

Supplementary Figure 2. (a) $C_{60}ZnI_2$@MWCNTs and **(b)** $C_{60}CeI_3$@MWCNTs samples after washing with distilled water. **(c)** Open ended MWCNTs showing the presence of fullerenes in its interior after the C_{60} corking of filled MWCNTs (black arrow); external fullerenes can also be observed (white arrows).

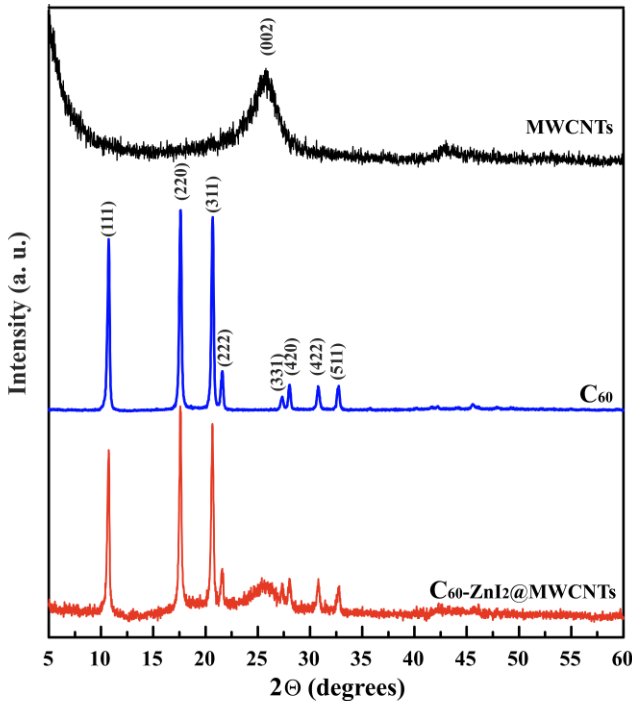

Supplementary Figure 3. X-ray diffraction patterns of MWCNTs (continuous black line), C_{60} (continuous blue line) and $C_{60}ZnI_2$@MWCNTs (continuous red line) measured in the range 2θ= 5-60°.

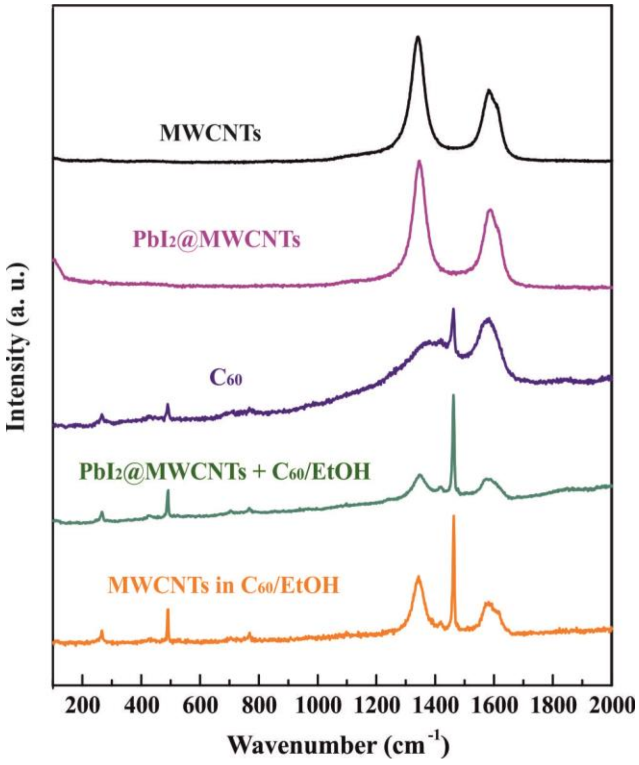

Supplementary Figure 4. Raman spectra of empty nanotubes (MWCNTs, black continuous line), C_{60} (blue continuous line), MWCNTs treated in presence of a C_{60}/EtOH mixture (orange continuous line) and PbI_2@MWCNTs before (pink continuous line) and after (green continuous line) washing with the C_{60}/EtOH mixture. Spectra were acquired with a 532 nm laser excitation.
